# Nutritional Ergogenic Aids in Racquet Sports: A Systematic Review

**DOI:** 10.3390/nu12092842

**Published:** 2020-09-17

**Authors:** Néstor Vicente-Salar, Guillermo Santos-Sánchez, Enrique Roche

**Affiliations:** 1 Biochemistry and Cell Therapy Unit, Institute of Bioengineering, University Miguel Hernandez, 03201 Elche, Spain; eroche@umh.es; 2 Department of Applied Biology-Nutrition, Alicante Institute for Health and Biomedical Research (ISABIAL-FISABIO Foundation), University Miguel Hernandez, 03201 Elche, Spain; 3 Departamento de Tecnología de la Alimentación y Nutrición, Universidad Católica de Murcia, 30107 Murcia, Spain; gsantos-ibis@us.es; 4 CIBER Fisiopatología de la Obesidad y Nutrición (CIBEROBN), Instituto de Salud Carlos III (ISCIII), 28029 Madrid, Spain

**Keywords:** racquet sports, ergogenic aid, performance, sport supplement

## Abstract

A nutritional ergogenic aid (NEA) can help athletes optimize performance, but an evidence-based analysis is required in order to support training outcomes or competition performance in specific events. Racquet sports players are regularly exposed to a high-intensity workload throughout the tournament season. The activity during a match is characterized by variable durations (2–4 h) of repeated high-intensity bouts interspersed with standardized rest periods. Medline/PubMed, Scopus, and EBSCO were searched from their inception until February 2020 for randomized controlled trials (RCTs). Two independent reviewers extracted data, after which they assessed the risk of bias and the quality of trials. Out of 439 articles found, 21 met the predefined criteria: tennis (15 trials), badminton (three trials), paddle (one trial), and squash (two trials). Among all the studied NEAs, acute dosages of caffeine (3–6 mg/kg) 30–60 min before a match have been proven to improve specific skills and accuracy but may not contribute to improve perceived exertion. Currently, creatine, sodium bicarbonate, sodium citrate, beetroot juice, citrulline, and glycerol need more studies to strengthen the evidence regarding improved performance in racquet sports.

## 1. Introduction

Racquet sports are included in the family of ball sports and more specifically, among those using an implement. They are characterized by the use of a manual racquet to propel an implement (a ball, shuttlecock, etc.) between two or four players with the objective of placing it in a position with no return possibilities for the opponent. There are two different game formats: (a) passing the implement over a net in a divided field (tennis, badminton, paddle and table tennis) or (b) hitting the implement onto a wall in a shared field (squash and racquetball) [1].

Racquet sports are acyclic disciplines with very intense workload cycles, which are interrupted by small pauses that allow for an incomplete recovery. Therefore, metabolic demands in racquet sports alternate between both anaerobic and aerobic energy sources. Anaerobic energy comes from intramuscular ATP and phosphocreatine (PC), as well as from anaerobic glycolysis, the three of which are used during high intensity, short duration points, changes of direction, and hits. On the other hand, the aerobic system is involved during long points of moderate intensity, playing a primary role in delaying fatigue, and indirectly, favoring concentration, technical skills, and maintaining workload during a match [2,3,4,5].

As a result of this fact, the average heart rate (HR) during a match reaches up to 60–80% of HR maximum (HRmax), increasing to 90% of HRmax in high-intensity situations [6,7,8]. Nonetheless, HRmax does not provide clear information regarding real energy demands or the metabolic pathways involved, since this parameter is affected by dehydration, heat stress, age, and playing techniques [9]. Measuring blood lactate concentration during a match could report more accurately the energetic pathways used by racquet sports players. Ranges vary from 1.0–4.0 mmol/L to 8.0–12.0 mmol/L during prolonged high-intensity matches [2,10,11,12], supporting the key role of glycolytic pathways during the match.

An ergogenic aid is any training method, mechanical device, nutritional or pharmacological approach, or psychological technique that can improve exercise performance capacity and/or improve training adaptations [13]. Therefore, a nutritional ergogenic aid (NEA) is defined as those nutritional supplements taken orally containing a nutritional ingredient that intends to complement diet. The objective of these supplements is to improve sports performance without exerting harmful effects on the individual [14].

The consumption of NEAs has been increasing in recent years around the world, which has led to a great variety of research with the aim of estimating their intake and use. In fact, sales of dietary supplements grew 6.1% in 2017, achieving an income of 39.8 billion dollars in the US [15]. A meta-analysis published in 2015 concluded that elite athletes used many more dietary supplements than non-elite athletes, and the prevalence of use was similar in men and women [16]. The NEAs most frequently used by high-level tennis players tend to be creatine and caffeine [17] while among international rank squash players, sodium bicarbonate is also frequently consumed in addition to the two aforementioned NEAs [18]. Normally, NEA recommendations in high-level racquet sports players are directed by personal trainers, coaches, or sports dietitian–nutritionists. However, proper counseling based on current scientific evidence is required.

In this line, several organizations such as the Australian Institute of Sport (AIS) or the World Anti-Doping Agency (WADA) propose classifications of sports supplements grouped into different categories according to effectiveness, legality, and safety. Nevertheless, there are not policies regarding the regulation of alleged benefits and safety claims [19,20]. Thus, athletes find themselves under the influence of companies’ advertising, which claims improved performance and recovery through the consumption of a wide range of products without scientific evidence regarding their effect, dosage, or instructions for use.

The main aim of this systematic review was to evaluate the scientific evidence concerning NEAs in the improvement of performance of racquet sports athletes specifically through published RCTs.

## 2. Materials and Methods

The conduct and reporting of the current systematic review conform to the Preferred Reporting Items for Systematic Reviews and Meta-Analyses (PRISMA) [21]. Five racquet sports were analyzed regarding the effectiveness of certain nutritional ergogenic aids: tennis, badminton, squash, table tennis, and paddle.

### 2.1. Systematic Search

Relevant articles were identified by title and abstract in the electronic databases Medline, Scopus, and EBSCO (since inception to 20 February 2020) using the search strategy in Table 1. The electronic search was supplemented by a manual review of reference lists from relevant publications and reviews to find additional publications on the subject.

### 2.2. Data Extraction

Two reviewers (N.V.-S. and G.S.-S.) independently extracted the following data from each study using a predefined Microsoft Excel data extraction form including the number of participants within each group, participant characteristics, racquet sport discipline, and supplementation intervention characteristics, end points, measurement methods, and results in order to produce an overview table of all eligible studies.

### 2.3. Study Selection

Studies were eligible for inclusion if they met each of the following criteria: (a) not using any doping substances established by the World Anti-Doping Agency (WADA), (b) using a randomized controlled trial (RCT) design that included one group taking supplementation and 1+ groups receiving a placebo or not taking supplementation, (c) not including any ergogenic aids classified within group A by the Australian Sports Commission (AIS) because of their high evidence grade [22], (d) not presenting supplementation as a source of nutrients, such as bars, gels, or drinks rich in carbohydrates and electrolytes, and (e) not being gray literature (abstracts, conference proceedings, or editorials) or reviews.

### 2.4. Quality Assessment and Publication Bias

Characteristics of the retrieved RCTs were evaluated using the ‘risk-of-bias’ assessment tool following the recommendations by the Cochrane Handbook for Systematic Reviews of Interventions [23,24]. This evaluation was carried out by two reviewers (N.V.S. and G.S.S.) working independently in order to present bias comprehensively. The following criteria were analyzed: randomized treatment order and carry-over effect (selection bias), blinding of participants and research staff to group allocation (performance bias), blinding of outcome assessor (detection bias), incomplete outcome data (attrition bias), selective reporting (reporting bias), and other bias (it was assessed if there was controlled diet, exercise use of supplements or drugs, and sport stratification when a mixture of disciplines was analyzed). Then, the retrieved RCTs were classified as being of “high”, “unclear”, or “low” risk of bias. Effect size was calculated using Cohen´s d test.

## 3. Results

### 3.1. Included Studies

**Figure 1:**
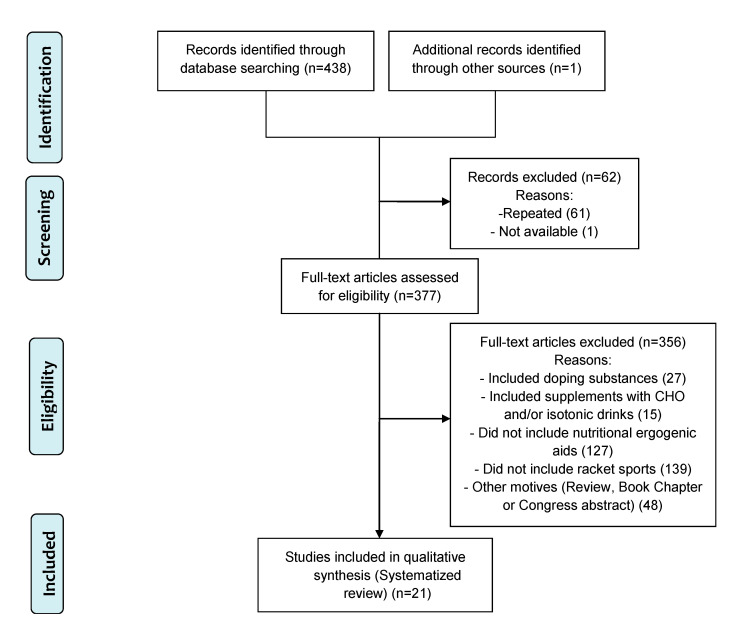
Preferred Reporting Items for Systematic Reviews and Meta-Analyses (PRISMA) flow chart [21] of the study selection process.

A total of 438 studies were screened by title and abstract, and 377 were assessed for eligibility criteria (full-text screening). From the retrieved articles, twenty-one met all the inclusion criteria and were included in the systematic review (Figure 1, Table 2 and Table 3). Thirteen RTCs were found in the Medline database (eleven for tennis and two for badminton), seven were found in the Scopus database (three for tennis, one for badminton, two for squash, and one for paddle) where one article was not available despite requesting it from its main author; and none were retrieved from the EBSCO database (because all those found there were repeated). Additionally, one article that was not found through the initial search but was found in a review published in the Medline database was added for full-text analysis. The PRISMA flowchart was applied to illustrate the step-by-step exclusion of unrelated/duplicate retrieved records, leading to the final selection of twenty-one RCTs that met the predefined inclusion criteria (Figure 1).

### 3.2. Risk of Bias and Quality Assessment of Studies

The risk of bias of the included studies is illustrated in Table 4. The RCTs of Pluim; 2006 [33] and Hartono; 2017 [42] were parallel group trials, so the criteria of random sequence generation and allocation concealment were used instead of randomized treatment order and carry-over effect respectively. Most of the trials assessed showed an unclear level in the criteria of selection bias, both in the randomized treatment order and in the evaluation of the carry-over effect. Only the trials by Vergauwen; 1998, [26] Lopez-Samanes; 2020 [36] and Abian; 2015 [40] suitably described the tools used for randomization treatment, while trials by Wu; 2010 [34], Yang; 2017 [38], Abian; 2015 [40] and Muller; 2019 [45] used tests to check if the washout time between conditions was suitable. Moreover, most of the studies also showed a high risk of detection bias except for trials by Gallo-Salazar; 2015 [30] and Abian; 2015 [40], which specifically indicated that blinding was kept until the statistical analysis was performed. Five studies [27,38,42,44,45] were at a high risk of performance bias due to incomplete blinding or a lack of blinding.

### 3.3. Participants

Age in all the examined studies ranged from 16.4 to 51.0 years old, so that included from junior to master players. Level ranged from university to professional level in both sexes, with a majority of players being males (*n* = 266) as compared to females (*n* = 27). Tennis was the racquet sport about which more studies on NEAs were checked (*n* = 15), followed by badminton (*n* = 3), squash (*n* = 2), and paddle (*n* = 1), but no study was found for table tennis. In the case of NEAs, caffeine was the most evaluated supplement (*n* = 10) followed by creatine monohydrate (*n* = 4), plasma buffers (*n* = 3), nitric oxide (NO) precursors (*n* = 3), and hydration agents (*n* = 1).

### 3.4. Nutritional Ergogenic Aids and Intervention Characteristics in Tennis

Caffeine was the most tested NEA with seven studies (Table 2). All trials had a duration of 1 day with variations in concentrations and timing. Most studies selected used a caffeine dosage of 3–6 mg/kg 30–60 min before the tests [26,27,28,30,31], with improvements in specific tennis skills such as accuracy serve, backhand stroke, serve velocity in last sets, total number of successful shots, handgrip force, and number of sprints compared with control groups. Other protocols with continued administration during tests but a smaller dosage (0.2–0.25 mg/kg) [25] or the same quantity of caffeine given to each player (80 mg) [29] only show an increase in urine epinephrine or no changes compared with control groups respectively.

Regarding creatine monohydrate, only two studies evaluated its efficiency. Neither a high dosage for five days (20 g/day) [32] nor a load period of six days (0.3 g/kg) followed by a maintenance period (0.03 g/kg) until completing five weeks [33] offered advantages compared to control groups.

NEAs related to plasma buffer function were evaluated by two one-day duration studies. A load of 0.3 g/kg sodium bicarbonate 70 min before test and continuous intake of 0.1 g/kg during test showed maintenance of serve and stroke consistency (number of balls landed within the singles court on the designated side) compared to the control group [34], while 0.5 g/kg sodium citrate 120 min before test increased stroke consistency [35]. Both NEAs increased plasma lactate significantly, but only sodium citrate was accompanied by an increase in blood pH.

Three studies about NO precursors were found. The intake of 70 mL beetroot juice 3 h before the test did not show differences compared to control [36]. On the other hand, only citrulline malate supplementation (80 g 60 min before the trial) [37] or together with arginine and BCAAs (0.05 g/kg 80 min before test) [38] showed improvements compared with control. Citrulline malate improved handgrip strength and relative peak power, and citrulline + arginine + BCAAs avoided the decrease of stroke accuracy and kept stroke consistency and stroke velocity.

Lastly, regarding hydration agents, glycerol was the only NEA found in just one study [39]. The consumption of 1 g/kg glycerol 150 min before the trial and 0.5 g/kg 15 min after it increased body weight, plasma osmolality, and plasma volume, and decreased urine volume.

### 3.5. Nutritional Ergogenic Aids and Intervention Characteristics in Badminton, Squash, and Paddle

Caffeine was tested in badminton male players 60 min before exercise protocol with a dosage ranging from 3 to 4 mg/kg [40,41]. It showed improvements in jumps and the number of impacts accompanied with a decrease in errors in anticipation, reaction time, and time of sprints (Table 3). In addition, in paddle, caffeine showed ergogenic effects. The intake of 6 mg/kg caffeine 30 min before the exercise protocol in twelve amateur male players increased the percentage of correct hits, diminishing errors [45].

Different plasma buffers were evaluated by one study in badminton male players [42]. A load of 0.3 g/kg sodium bicarbonate or 0.3 g/kg sodium citrate 90 min before the test showed an increase in time to exhaustion with both supplements (51.3 and 44.4% respectively). Both NEAs increased plasma lactate, but only sodium bicarbonate showed an increase in plasma pH.

Finally, the effect of creatine was evaluated in squash players by two studies, one of them with a load of 0.3 g/kg/day for 5 days before test [43], and the other with acute supplementation of a mixed product composed by 1 g creatine + 1.5 g guarana + 133 mg caffeine [44]. The creatine loading protocol showed a decrease in sprint time, while acute supplementation with guarana and caffeine increased peak power and decreased fatigue, reaction time under pressure, and time visual response.

## 4. Discussion

### 4.1. Effects of Caffeine in Racquet Sports

Caffeine has shown to be an effective ergogenic aid for aerobic and anaerobic exercise with improvements in performance and the perceptions of exertion and muscle pain with dosage ranging from 2.35 to 5 mg/kg [46,47]. A similar dosage range was used in most of the racquet sports studies that showed positive effects and a low risk of bias [26,30,40,41] with the exception of Hornery; 2007 [27] due to its methodology of randomization and blinding. Even using higher doses (6 mg/kg), the positive effects are verified [28,31,45] but with a moderate risk of bias due to aspects of randomization or blinding.

In tennis, caffeine improved power skills such as backhand stroke, serve velocity, handgrip force, and the number and velocity of sprints, as well as mental aspects such as the accuracy serve or total number of successful shots. Lower dosages such as 0.2–0.25 mg/kg before and during a tennis match or approximately 1 mg/kg 30 min before a serve test only increased epinephrine levels in urine but they have not shown any performance improvements [25,29]. Moreover, both studies have a moderate risk of bias since the control of randomization, the carry-over effect, the differences in caffeine dosage between sexes, and the lack of certain control groups could be affecting the results. In another study [26], it was shown that the use of a carbohydrate drink with or without caffeine showed improvements in sprints and serve quality compared with the placebo group. As there were no differences between both conditions, it is not possible to evaluate the real effect of caffeine in this study. Furthermore, although an increase in sweat rate has been observed with low caffeine dosages (3 mg/kg) in junior tennis players [30], several studies have disproved a dehydration risk [14].

On the other hand, handgrip force was not affected in badminton and paddle [40,45], but squat and counter jump height or power were significantly better than in the placebo group in badminton [40], offering a specific advantage in this discipline due to the net height in this sport.

In short resistance training, positive results have been observed regarding caffeine consumption in the reduction of perceived exercise exertion [47]. However, no changes were observed in racquet sports (long duration intermittent sports) with the only exception of using two intakes of 4 mg/kg caffeine before and after the first half of a badminton specific test and combining them with carbohydrates [41]. Despite these null effects, the number of total successful shots (with medium effect size (d = 0.57)) and volley precision (high effect size (d = 0.86)) were improved in tennis and paddle respectively using a high caffeine dosage (6 mg/kg) [28,45].

Therefore, the use of an acute ergogenic dosage of caffeine (3–6 mg/kg) 30–60 min before a match is better than the intake of smaller concentrations, despite its continuous use during the match. Due to the large seasons with accumulative long duration matches such as tennis, caffeine consumption could be a useful aid for all competitive levels, since it may maintain physical and mental conditions. More studies with a high caffeine dosage during long periods of intermittent exercise and in combination with carbohydrates are needed in order to prove caffeine capacity to elicit high accuracy and synergetic effects.

### 4.2. Effects of Creatine Monohydrate in Racquet Sports

Commonly, creatine monohydrate supplementation has been used as a strategy to increase muscle mass and strength during training, but it has been also reported to improve power and anaerobic capacity [48,49,50]. Thus, the use of creatine in intermittent sports such as racquet sports is of high interest since about 75% of top 100 rank tennis players take it [17]. However, up to the present, there is no evidence for recommendation.

Neither specific tennis skills, such serve or stroke, nor general physical aptitudes, such as sprints or strength (typical short-duration high-intensity movements), were improved with different protocols involving only a creatine load (20 g/day for 5 days) or load and maintenance (0.3 g/day for 6 days and 0.03 g/kg for 28 days) [32,33]. Both creatine protocols were used in two studies that had a low to moderate risk of bias.

On the contrary, in one study on squash, the intake of 0.3 g/kg creatine for 5 days was capable of improving the sprint time in a specific test on court [43]. Due to the heterogeneity of sprint protocols, it is not possible to reach a solid conclusion regarding its effect and the effect of moderate bias due to lack of information about the randomization method, carry-over effect and missing information about placebo composition. Moreover, the combination of 1 g of creatine + 1.5 g of guarana + 133 mg of caffeine in an acute dosage improved several physical and alertness aptitudes in squash players, but it has not been ruled out that the stimulant effect of guarana and caffeine were behind them [44]. Therefore, this fact—together with the lack of blinded groups—led to a high risk of bias.

Further studies should evaluate the sprint capacity or service and stroke skills with a high dosage (16 g/day or 0.3 g/kg/day) for a longer period (at least 14 days), as has been shown in previous works [49,50]. Further studies should also consider protocols that emulate long games. In addition, despite not showing any clear improvements in specific tennis skills, creatine consumption during the pre-season could be beneficial for the maintenance or increase of lean mass [50].

### 4.3. Effects of Buffering Supplements in Racquet Sports

High-intensity intermittent exercise tends to accumulate acid (H^+^) and carbon dioxide (CO_2_) in the muscle and blood. Bicarbonate coming from CO_2_ acts as the primary mechanism to counteract plasma acidification. The efficiency of acute sodium bicarbonate supplementation is influenced by exercise duration. Specifically, extended duration (>4 min) sports have shown diverse results, with sodium bicarbonate improving performance in running and cycling, but not in rowing, rugby, water polo, or basketball [51].

An acute dosage (0.3 g/kg) and a continuous intake for a >4 min specific tennis test (0.1 g/kg) did not improve accuracy or perceptual exercise exertion but kept serve (small effect size (d = 0.42)) and stroke consistency (small effect size (d = 0.09)), which decreased in placebo condition [34] with a low risk of bias. On the other hand, in badminton players, only an acute dosage of 0.3 g/kg increased time to exhaustion in a treadmill test, but not in a specific test in a high risk of bias study, since the randomized method, allocation concealment, blinding method and control of diet, other supplementation consumption, and exercise load were poorly controlled [42]. Although the blood lactate level was higher than in the placebo groups in both studies, this could be due to the carboxylate co-transporter, which extracts lactate and H^+^ from working muscle cell to circulation after an increase in extracellular pH [52] and an increase of glycolytic activity. Despite no changes observed in extracellular pH between placebo and sodium bicarbonate before and after tests in tennis players [34], changes between pre- and post-tests with supplementation may be enough to activate lactate extrusion due to an enhance of glycolytic metabolism.

Other buffer supplements such as sodium citrate, used for causing less gastrointestinal distress than other supplements, also showed significant high values of blood lactate compared to placebo after an acute dosage (0.3–0.5 g/kg) 90–120 min before exercise [35,42]. Nevertheless, an increase in extracellular pH was observed in tennis players, [35] which decreased in badminton players [42], but there are contradictions about it, since the text of the study indicates otherwise. On the other hand, sodium citrate was able to increase stroke consistency (high effect size (d = 1.41)) in junior tennis players, just as sodium bicarbonate did, but it did not present effects in accuracy and perceptual exercise exertion in protocols of >4 min duration [35]. With a non-specific badminton test, sodium citrate was able to improve the time to exhaustion in a treadmill test [42]. Both studies have a moderate to high risk of bias due mainly to the blinding methodology and the control of the intake of other supplementation.

More studies with a higher number of subjects would be needed with the aim of achieving strong evidence about improvements in tennis skills as well as evidencing possible synergies between different buffers (for example, beta-alanine) and other NEAs.

### 4.4. Effects of Nitric Oxide (NO) Precursors in Racquet Sports

It is well known that NO plays a relevant role as a second messenger. Its production is also related to an increase in blood flow, which enhances nutrient and hormone delivery. NO also has a favorable impact on resistance and endurance training adaptations [53,54]. Recent systematic reviews and meta-analysis about NO synthase-independent pathway supplements showed that potassium nitrate and sodium nitrate were less effective than beetroot juice on endurance exercise. The use of beetroot juice supplementation containing 12–6 mmol nitrate displayed significant improvements in time to exhaustion in a cycling race of 5–30 min duration but slightly non-significant improvements in time trial or graded-exercise performance [55,56].

In intermittent sports such as tennis, beetroot juice containing 6.4 mmol nitrate did not show any improvements in either explosive movements (serve velocity, jump, sprint, handgrip force) or perceptual exertion in high-level tennis players [36] with a low risk of bias. These results are similar to the ones found in recent studies in which short and high-intensity movements (such as countermovement jump, isometric strength, or muscular movement concentric velocity) were evaluated after the consumption of beetroot juice containing 6.4–17.7 mmol nitrate [57,58,59]. It seems that the effect of beetroot juice could be beneficial in endurance performance due to nitrate conversion to NO, affecting improvement in aerobic adenosine triphosphate (ATP) synthesis due to a reduction of VO_2_. In intermittent and short-term exercise, where the anaerobic alactic system is the main source of energy, the effects are less clear. Only one-third of the studies evaluated in a recent systematic review of intermittent exercise protocols [60] showed significant results in different variables of power compared with the placebo group during repeated-sprint tests.

On the other hand, NO synthase-dependent pathway supplements, such as arginine or citrulline, have shown different results. While arginine supplementation has demonstrated improvements in both aerobic and anaerobic performance with acute (0.15 g/kg) or chronic (1.5–2.0 g/day for 4–7 weeks or 10–12 g/day for 8 weeks) protocols [61], acute protocols of citrulline supplementation (3–6 g) showed a small effect size (0.2) on high-intensity strength and power performance in resistance exercise [62]. In master female tennis players (51.0 ± 9.0 years), acute protocol with 8 g of citrulline improved handgrip strength and power peak in a specific anaerobic test, but not the capacity of sustained power or jump power [37]. Due to the lack of a washing time between conditions and control of the consumption of other stimulant substances, the risk of bias is moderate. Further studies are necessary to analyze the role of citrulline supplementation in the performance of younger racquet sports players.

Yang et al. (2017) [38] showed improvements regarding the prevention of a decrease in stroke accuracy and keeping stroke consistency and velocity (as opposed to a worsening in the placebo group) using 0.05/kg citrulline +0.05 g/kg arginine +0.17 g/kg branched-chain amino acids (BCAAs). The study presented a low risk of bias. Additionally, perceived exertion after the test decreased significantly. These results appear to be due to a lower plasma tryptophan/BCAAs ratio than placebo, since theoretically, BCAAs compete for the same tryptophan transporter across the blood–brain barrier, avoiding serotonin formation and, consequently, central fatigue instauration [63]. It is common to use a mixture of several NEAs in one product with the objective to obtain a synergic effect, but further studies are necessary in order to verify the true effects of citrulline or arginine by themselves, without the presence of the BCAAs being able to distort them.

### 4.5. Effects of Glycerol Supplementation in Racquet Sports

Finally, glycerol is a naturally occurring metabolite that acts as a plasma expander and could help athletes prevent dehydration and improve thermoregulatory and cardiovascular changes [14]. Until 2018, the World Anti-Doping Agency (WADA) considered glycerol a banned substance, since it was hypothesized that it may alter athlete biological passport [64]. In any case, the results of its supplementation are mixed both in endurance and anaerobic disciplines [14]. In intermittent sports such as tennis, 1.0 g/kg glycerol before followed by 0.5 g/kg after 75 min of simulated match, in environmental conditions in the range of 29–38 °C and 50–90% relative humidity (emulating conditions of important tennis tournaments such as The Australian Open Grand Slam or Miami ATP Masters 1000), was not capable of improving accuracy in serves or strokes, sprint velocity, or agility, in spite of its effect increasing pre- and post-exercise plasma volume and osmolality [39]. This study has a moderate risk of bias, since its randomized method, carry-over effect, blinding method and control of diet, and other supplementation and drug consumption were poorly controlled. More research is needed to determine glycerol’s supposed potential efficacy in racquet sports during more time-prolonged matches or during several matches on the same day or on consecutive days in hot conditions.

## 5. Conclusions

Caffeine is the NEA showing clearer evidence of benefits for racquet sport players. Acute dosages (3–6 mg/kg) 30–60 min before a match may improve specific skills and accuracy but may not contribute to improve perceived exertion. Even though some evidence concludes that other NEAs, such as creatine, sodium bicarbonate, sodium citrate, beetroot juice, citrulline and glycerol, could play an interesting role in improving performance, more studies are needed to strengthen the evidence (Table 5).

## Figures and Tables

**Table 1:** Combined terms used in the search for studies in the database. ^1^ Mesh terms were used in the search; ^2^ Term not included in the Mesh search; ^3^ nutritional ergogenic aid (NEAs) filed in the A group of the Australian Institute of Sport (AIS).

Pubmed ^1^			Scopus and EBSCO		
NEA		Sport	NEA ^3^		Sport
Dietary supplements	**AND**	Racquet Sports	Dietary supplements	**AND**	Racquet Sports
Caffeine		Tennis	Ergogenic aid		Tennis
Creatine			Caffeine		Badminton
Beta-alanine			Creatine		Table tennis
Sodium Bicarbonate			Beta-alanine		Squash and sport
Ergogenic aid ^2^			Sodium Bicarbonate		Paddle
			Nitrate		
			Beetroot juice		
			Glycerol		

**Table 2:** Included studies on nutritional ergogenic aids in tennis. BCAAs: Branched-chain amino acids; FFA: Blood free fatty acids; Glu: Blood glucose; Gly: Blood glycerol; HR: Heart rate; Lac: Blood lactate; LTPT: Leuven Tennis Performance Test; LTST: Loughborough Tennis Skill Test; NO; Nitric oxide; Pl: Placebo; RSA: repeated-sprint ability shuttle test; STPT: Skill Tennis Performance Test; Trp/BCAAs: Blood tryptophan/branched-chain amino acids ratio; u-EPI: Urine epinephrine; u-NE: Urine norepinephrine. ↑: Significant increase compared to placebo/control group; ↓: Significant decrease compared to placebo/control group; ↔: without changes compared to placebo/control group.

Study	NEA	Dosage/Time	Participants	Age (yrs)	Level	Blinded/Double Blinded	Duration	Exercise Protocol	Measurements	Main Outcomes
[25]	Caffeine	- 0.2 (women)–0.25 (men) mg/kg/0 min before match and every 15 min during a match	16 (8 men/8 women)	25.4 ± 1.9/20.4 ± 2.8	National ranking (Germany)	DB	1 day	3 matches (2 of 75 min/match and 1 of 90 min/match with only rest between match 2 and 3 of 30 min) + Accuracy and sprint test	- Lac	↔ Lac
									- Glu	↔ Glu
									- Gly	↔ Gly
									- FFA	↔ FFA
									- u-EPI	↑ u-EPI
									- u-NE	↔ u-NE
									- Sprints	↔ Sprints
									- Accuracy hit	↔ Accuracy hit
									- Perceived exertion	↔ Perceptual training intensity
[26]	Caffeine	- 5 mg/kg/60 min before pre-test. - 0.75 mg/kg/Each 1 h after start pre-test and during protocol	13 men	20.4 ± 0.9	National ranking (Belgium)	DB	1 day	LTPT + Sprint test + Court session (120 min) + LTPT	- Sprints	↔ Sprints
									- Serve quality	↔ Serve quality
									- Backhand stroke quality	↑ Backhand stroke
									- Volley errors and fatigue	↑ Volley errors and fatigue
									- HR	↔ HR
									- Perceived exertion	↔ Perceptual training intensity
[27]	Caffeine	- 3 mg/kg/30 min before match	12 men	18.3 ± 3.0	National ranking (Australia)	B	1 day	1 match of 160 min/match	- Lac	↔ Lac
									- Glu	↔ Glu
									- CK	↔ CK
									- Prolactin	↔ Prolactin
									- Fluid loss	↔ Fluid loss
									- Serve and stroke velocity	↑ Serve velocity in 4th set
									- Serve kinematics	↔ Serve kinematics
									- Perceptual skills	↔ Perceptual skills
									- HR	↔ HR
									- Perceived exertion	↔ Perceptual training intensity
[28]	Caffeine	- 6 mg/kg/60 min before test	16 (8 men/8 women)	20.7 ± 1.7	National ranking (USA) University players (UK)	DB	1 day	Intermittent treadmill exercise (45 min) + Tennis skills test	- Successful shots	↑ Total shot successes
									- HR	↔ HR
									- Perceived exertion	↔ Perceptual training intensity
[29]	Caffeine	- 80 mg/30 min before test	12 (6 men/6 women)	18–22		DB	1 day	3 days of sleep restriction follow a day of accuracy serve test	- Accuracy serve	↔ Accuracy serve
[30]	Caffeine	- 3 mg/kg/60 min before test	14 (10 men/4 women)	16.4 ± 1.2	Elite-level Junior players (Spain)	DB	1 day	Tennis specific test + Simulated Match of best-of-3-sets system	- Handgrip force	↑ Handgrip force
									- Serve velocity	↔ Serve velocity
									- Running speed	↑ Only in high intensity
									- Number of sprints	↑ Number of sprints
									- Distance	↔ Distance
									- HR	↔ HR
									- Sweat rate	↑ Sweat rate
[31]	Caffeine	- 6 mg/kg/60 min before test	10 (5 men/5 women)	19.9 ± 1.8	National ranking (USA)	DB	1 day	- Tennis serve trial + Shuttle run sprint + Tennis serve trial	- Accuracy serve	↑ Accuracy serve (depending of conditions of time and distance
									- Shuttle run time	↔ Shuttle run time
									- Likert scale	↔ Feelings
[32]	Creatine	- 20 g/day (4 × 5g/day)/During 5 days before test	8 men	20.4 ± 0.9	National ranking (Belgium)	DB	5 days	LTPT + Shuttle run sprint	- Quality of 1st and 2nd service	↔ Service quality
									- Stroke quality	↔ Stroke quality
									- Sprint power	↔ Sprint power
[33]	Creatine	- 0.3 g/kg in loading phase (6 days) - 0.03 g/day in maintenance phase (28 days)	36 men	22.5 ± 4.9–28.8 ± 4.8	ITN 3	DB	5 weeks	- Service test + Ball machine ground stroke drill + Intermittent sprint test + Strength test	- Lac	↔ Lac
									- Serving velocity	↔ Serving velocity
									- Stroke velocity	↔ Stroke velocity
									- Sprinting velocity	↔ Sprinting velocity
									- Strength	↔ Strength
									- HR	↔ HR
									- Perceived exertion	↔ Perceptual training intensity
[34]	Sodium Bicarbonate	- 0.3 g/kg/70 min before test - 0.1 g/kg/During test	9 men	21.8 ± 2.4	College Tennis players (Taiwan)	DB	1 day	- LTST + Simulated match (50 min) + LTST	- Lac	↑ Lac
									- pH	↔ pH
									- Serve consistency	Keeps serve consistency while Pl ↓
									- Stroke consistency	Keeps stroke consistency while Pl ↓
									- Serve Accuracy	↔ Serve Accuracy
									- Stroke Accuracy	↔ Stroke Accuracy
									- HR	↔ HR
									- Perceived exertion	↔ Perceptual training intensity
[35]	Sodium Citrate	- 0.5 g/kg/120 min before test	10 men	17.0 ± 1.0	Junior National Ranking (Brazil)	DB	1 day	STPT + RSA + Simulated match (60 min) + STPT + RSA	- Lac	↑ Lac
									- pH	↑ pH
									- Stroke consistency	↑ Stroke consistency
									- Stroke accuracy	↔ Stroke accuracy
									- Number strokes	↔ Number strokes
									- Time of sprints	↔ Time of sprints
									- Perceived exertion	↔ Perceptual training intensity
[36]	Beetroot juice	- 70 mL (6.4 mmol of NO^3−^)/3 h before test	13 men	25.4 ± 5.1	ATP and National ranking (Spain)	DB	1 day	- Serve velocity test + Counter movement jump + Isometric handgrip strength + Agility and sprint test	- Serve velocity	↔ Serve velocity
									- Jump height	↔ Jump height
									- Handgrip force	↔ Handgrip force
									- Agility	↔ Agility
									- Sprint velocity	↔ Sprint velocity
									- Perceived exertion	↔ Perceptual training intensity
[37]	Citrulline-malate	- 8 g/60 min before test	17 women	51.0 ± 9.0	Masters ranking in USTA (USA)	DB	1 day	- Isometric handgrip strength + Counter movement jump + Wingate cycling test	- Handgrip force	↑ Handgrip strength
									- Peak vertical power	↔ Jump power
									- Anaerobic capacity	↔ Anaerobic capacity
									- Relative peak power	↑ Relative peak power
									- Explosive power	↑ Explosive power
									- Sustained power	↔ Sustained power
[38]	BCAAs + Arginine + Citrulline	- 0.17 g/kg BCAAs (Leu–Ile–Val = 10:7:3) + 0.05 g/kg Arginine + 0.05 g/kg Citrulline/80 min before test	9 men	25.6 ± 0.7	National Ranking (Taiwan)	B	1 day	Perceptual-motor performance test (LTST modified) + Simulated match (120 min) + Perceptual-motor performance test (LTST modified)	- Lac	↔ Lac
									- Gly	↔ Gly
									- Glu	↔ Glu
									- FFA	↔ FFA
									- NO	↑ NO
									- Trp/BCAAs	↓ Trp/BCAAs
									- HR	↓ HR
									- Stroke Accuracy	Prevents a high decrease in stroke accuracy compared with Pl
									- Stroke consistency	Keeps stroke consistency while Pl ↓
									- Stroke velocity	Keeps stroke velocity while Pl ↓
									- Perceived exertion	↓Perceptual training intensity
[39]	Glycerol	- 1 g/kg/150 min before test - 0.5 g/kg/15 min after test	11 men	27.0 ± 2.0	Ranking 4–5 in USTA (USA)	DB	1 day	Tennis specific test + Simulated match (75 min) + Tennis specific test	- Change in body	↑ Body weight vs. Pl
									Weight	
									- Plasma osmolality	↑ Plasma osmolality vs. Pl (only pre- and post-exercise)
									- Change in plasma	↑ Plasma volume vs. Pl (only pre- and post-exercise)
									volume	
									- Electrolytes	↔ Electrolytes
									- Urine volume	↓ Urine volume
									- Sprint velocity	↔ Sprint velocity
									- Agility	↔ Agility
									- Stroke accuracy	↔ Stroke accuracy
									- Serve accuracy	↔ Serve accuracy

**Table 3:** Included studies on nutritional ergogenic aids in badminton, squash, and paddle. Glu: Blood glucose; HR: Heart rate; Lac: Blood lactate. ↑: Significant increase compared to placebo/control group; ↓: Significant decrease compared to placebo/control group; ↔: without changes compared to placebo/control group.

Badminton										
Study	NEA	Dosage/Time	Participants	Age (yrs)	Level	Blinded/Double Blinded	Duration	Exercise Protocol	Measurements	Main Outcomes
[40]	Caffeine	- 3 mg/kg/60 min before test	16 men	25.4 ± 7.3	National ranking (Spain)	DB	1 day	Handgrip force + Jump tests + Agility Test + Simulated match (45 min)	- Handgrip maximal force	↔ Handgrip force
									- Smash jump	
									- Squat jump	↔ Smash jump
									- Countermovement	↑ Squat jump height/power
									Jump (CJ)	↑ CJ height/power
									- Agility	↔ Agility
									- Number of impacts	↑ Number of impacts
									- HR	↔ HR
									- Perceived exertion	↔ Perceptual training intensity
[41]	Caffeine	- 4 mg/kg/60 min before exercise - 4 mg/kg /during 2nd Badminton specific test	12 men	28 ± 9	National ranking (United Kingdom)	DB	1 day	Badminton specific test + Fatigue protocol (33 min) + Badminton specific test	- Lac	↔ Lac
									- Glu	↔ Glu
									- Errors in anticipation	↓ Errors in anticipation
									- Accuracy serve	↔ Accuracy serve
									- Reaction time	↓ Reaction time
									- Time sprints	↓ Time sprints
									- HR	↔ HR
									- Perceived exertion	↓ Perceptual training intensity
[42]	Sodium bicarbonate	- 300 mg/kg/90 min before test	30 men	21	Student players (Indonesia)	?	1 day	Treadmill testing to exhaustion	- pH	↑ pH
									- Lac	↑ Lac
									- Time to exhaustion	↑ Time to Exhaustion
[42]	Sodium citrate	- 300 mg/kg/90 min before test	30 men	21	Student players (Indonesia)	?	1 day	Treadmill testing to exhaustion	- pH	↓ pH
									- Lac	↑ Lac
									- Time to exhaustion	↑ Time to Exhaustion
**Squash**										
[43]	Creatine	- 0.3 g/kg/day (4 × 0.075 g /kg/day)/during 5 days before test	9 (8 men/1 woman)	21.3 ± 0.3	National ranking (UK)	DB	5 days	Court set sprint test	- Lac	↔Lact
									- Sprint time	↓ Sprint time
									- Likert scale	↔ Feelings
									- HR	↔HR
[44]	Creatine + Guarana	- 1000 mg creatine + 1500 mg guarana + 133 mg Caffeine/Half dosage at 30 min and rest at 0 min before test.	8	18.2 ± 3.7	National ranking (France)	?	1 day	Cognitive tests + Cycle ergometer sprint test + Cognitive tests + Submaximal test with cognitive test	- Peak Power	↑ Peak power
									- Fatigue	↓ Fatigue
									- Reaction time	↔Reaction time
									- Reaction time under pressure	↓ Reaction time under time pressure
									- Visual response reaction time	↓ Visual response reaction time
									- Ocular motility response time	↓ Ocular motility response time
**Paddle**										
[45]	Caffeine	6 mg/Kg /30 min before test	12 men	27.7 ± 3.7	Amateur (Brazil)	B	1 day	Specific paddle training (45 min) + Handgrip strength and Volley test	- Isometric handgrip strength	↔ Handgrip strength
									- Volley precision	↑ % Correct hits
									- HR	↓ % Errors
									- Perceived exertion	↔ HR
										↔ Perceptual training intensity

**Table 4:** Quality assessment of the included studies. Cross-over studies where A = Randomized treatment order; B = Carry-over effect; C = Performance bias; D = Detection bias; E = Attrition bias; F = Reporting bias; G = Other bias. * Parallel studies where A = Random sequence generation and B = Allocation concealment.

Study	A	B	C	D	E	F	G
[25]	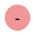	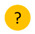	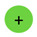	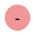	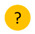	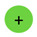	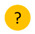
[26]	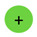	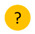	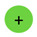	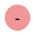	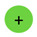	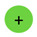	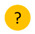
[27]	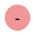	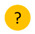	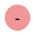	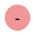	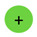	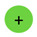	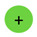
[28]	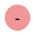	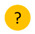	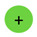	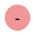	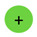	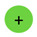	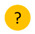
[29]	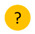	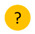	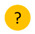	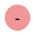	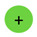	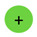	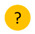
[30]	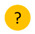	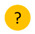	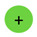	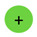	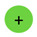	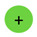	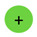
[31]	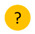	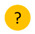	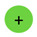	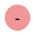	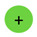	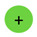	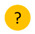
[32]	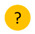	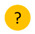	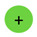	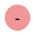	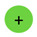	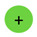	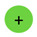
[33] *	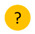	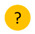	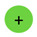	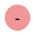	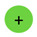	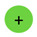	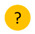
[36]	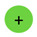	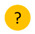	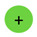	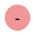	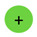	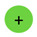	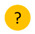
[37]	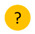	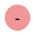	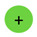	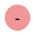	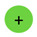	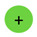	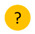
[39]	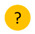	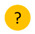	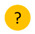	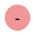	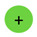	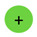	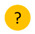
[34]	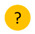	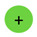	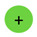	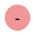	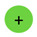	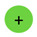	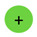
[35]	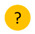	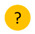	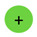	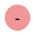	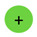	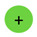	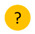
[38]	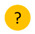	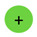	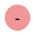	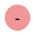	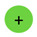	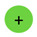	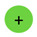
[40]	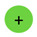	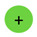	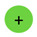	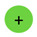	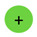	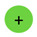	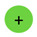
[41]	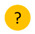	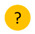	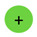	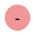	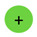	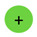	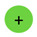
[42] *	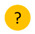	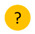	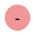	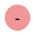	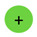	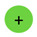	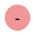
[43]	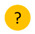	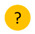	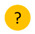	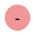	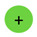	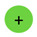	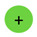
[44]	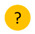	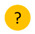	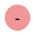	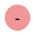	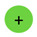	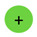	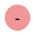
[45]	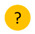	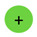	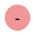	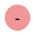	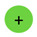	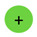	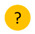
	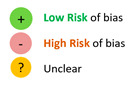						

**Table 5:** NEA recommendations from current evidence. Green: High level of recommendation due to the high number and quality of studies and the effects produced; Orange: Low level of recommendation due to the low number and/or quality of studies and the effects produced; Red: Not recommended due to the low number and quality of studies and contradictory or low effects.

NEA	Effects	Posology
Caffeine	- Improves specific racquet sports skills - Improves sprints and jumps - Improves mental performance and maybe accuracy	3–6 mg/kg 30–60 min before competition
Creatine	- May improve sprints	0.3 g/kg for 5 days
Sodium Bicarbonate	- May improve specific racquet sports skills - May hold up time to exhaustion	0.3 g /kg 70–90 min before competition
Sodium Citrate	- May improve specific racquet sports skills - May hold up time to exhaustion	0.3–0.5 g/kg 90–120 min before competition
Beetroot juice	- No effects	6.4 mmol 3 h before competition
Citrulline-malate	- May improve handgrip strength - May improve peak power	8 g 60 min before competition
Glycerol	- No effects	1 g/kg 150 min before competition and 0.5 g/kg 15 min after it.
